# Supplementary material for: The Optimized Preparation Conditions of Cellulose Triacetate Hollow Fiber Reverse Osmosis Membrane with Response Surface Methodology
Source: Polymers (Basel). 2023 Aug 28;15(17):3569. doi: 10.3390/polym15173569 (PMC10490516; doi:10.3390/polym15173569)
Supplement: Supplementary file 1 [file polymers-15-03569-s001.zip › polymers-2568907-supplementary.pdf]

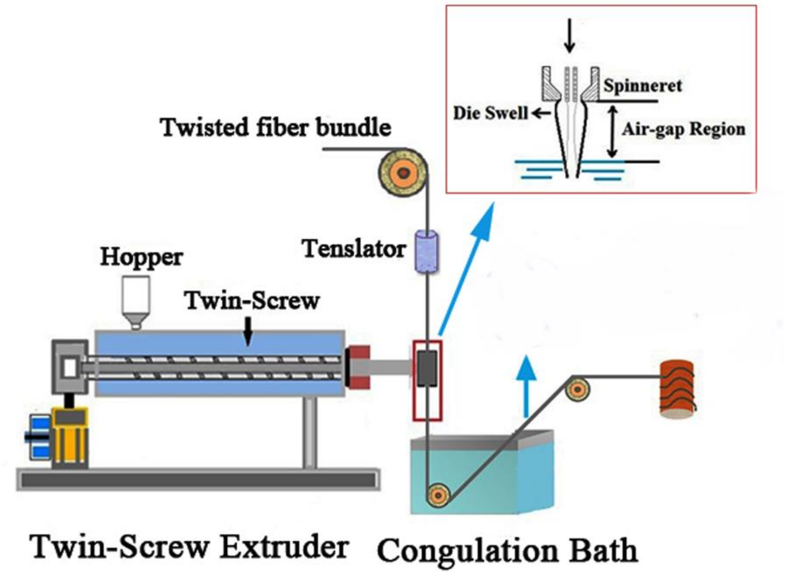

Figure S1. Schematic of RO membrane fabrication process [32].

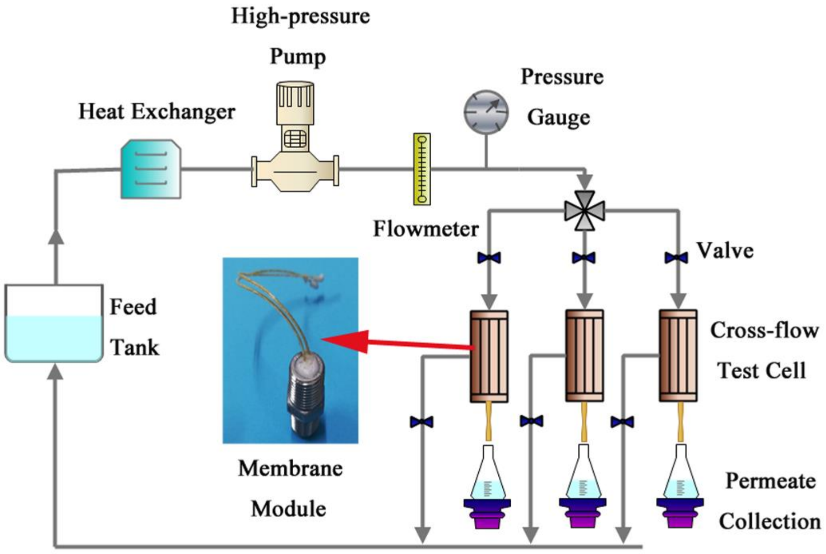

Figure S2. Schematic of evaluation RO membranes performance testing devices [32].
